# Supplementary material for: In Vitro Activity of Ebselen and Diphenyl Diselenide Alone and in Combination with Drugs against Trichophyton mentagrophytes Strains
Source: Pharmaceutics. 2022 May 28;14(6):1158. doi: 10.3390/pharmaceutics14061158 (PMC9229022; doi:10.3390/pharmaceutics14061158)
Supplement: Supplementary file 1 [file pharmaceutics-14-01158-s001.zip › pharmaceutics-1703457-supplementary.pdf]

Table S1. Minimal inhibitory concentrations (MIC; µg/ml) and fractional inhibitory concentration index (FICI) of interactions between antifungal agents and organoselenium compounds against clinical isolates of *Trichophyton mentagrophytes* – details

| Isolates | TRB+EBS | | | | TRB+DPDS | | | | ITC+EBS | | | | ITC+DPDS | | | | EBS+DPDS | | | |
|---|---|---|---|---|---|---|---|---|---|---|---|---|---|---|---|---|---|---|---|---|
| | $MIC_{comb}$ | | FICI | | $MIC_{comb}$ | | FICI | | $MIC_{comb}$ | | FICI | | $MIC_{comb}$ | | FICI | | $MIC_{comb}$ | | FICI | |
| TMH1/20 | 0.004 | 0.016 | 0.532 | I | 0.004 | 2 | 1.062 | I | 0.125 | 0.125 | 1.5 | I | 0.064 | 8 | 0.756 | I | 0.25 | 32 | 2 | I |
| TMH3/20 | 0.004 | 0.008 | 0.532 | I | 0.004 | 8 | 0.75 | I | 0.125 | 0.125 | 1.5 | I | 0.064 | 8 | 0.756 | I | 0.25 | 32 | 2 | I |
| TMH4/20 | 0.004 | 0.016 | 0,532 | I | 0.004 | 2 | 1.25 | I | 2 | 1 | 12 | A | 0.064 | 4 | 0.756 | I | 0.25 | 8 | 2 | I |
| TMH7/20 | 0.004 | 0.016 | 0.282 | S | 0.004 | 8 | 0.375 | S | 2 | 2 | 8 | A | 0.125 | 4 | 0.312 | S | 0.5 | 32 | 1.5 | I |
| TMH8/20 | 0.004 | 0.016 | 0.282 | S | 0.004 | 8 | 0.375 | S | 2 | 4 | 12 | A | 0.125 | 4 | 0.312 | S | 0.5 | 32 | 1.5 | I |
| TMH9/20 | 0.004 | 0.032 | 0.756 | I | 0.004 | 8 | 0.75 | I | 1 | 0.25 | 10 | A | 0.125 | 8 | 1.5 | I | 0.125 | 8 | 1.5 | I |
| TMH10/20 | 0.004 | 0.125 | 0.75 | I | 0.004 | 8 | 0.75 | I | 2 | 2 | 10 | A | 0.125 | 8 | 0.756 | I | 0.5 | 32 | 2 | I |
| TMH1/19 | 0.004 | 0.125 | 0.75 | I | 0.004 | 6 | 0.75 | I | 2 | 2 | 10 | A | 0.064 | 8 | 0.756 | I | 0.5 | 32 | 3 | I |
| TMH3/19 | 0.008 | 0.064 | 0.756 | I | 0.004 | 4 | 0.5 | I | 0.5 | 0.5 | 9.81 | A | 0.016 | 4 | 0.756 | I | 0.25 | 8 | 2 | I |
| TMH4/19 | 0.016 | 0.25 | 1 | I | 0.004 | 4 | 0.375 | S | 1 | 2 | 10 | A | 0.125 | 8 | 1 | I | 0.5 | 32 | 3 | I |
| TMH5/19 | 0.004 | 0.25 | 0.5 | S | 0.004 | 4 | 0.375 | S | 0.5 | 1 | 2 | I | 0.125 | 16 | 0.75 | I | 0.5 | 32 | 3 | I |
| TMH6/19 | 0.004 | 0.032 | 1.032 | I | 0.016 | 64 | 6 | A | 2 | 4 | 8 | A | 2 | 64 | 6 | A | 1 | 32 | 2 | I |
| TMH7/19 | 0.008 | 0.25 | 0.75 | I | 0.016 | 32 | 5 | A | 0.5 | 4 | 8 | A | 0.032 | 4 | 0.756 | I | 1 | 16 | 3 | I |
| TMH10/19 | 0.004 | 0.032 | 0.756 | I | 0.004 | 2 | 0.75 | I | 0.5 | 1 | 10 | A | 0.25 | 8 | 2 | I | 0.25 | 8 | 3 | I |
| TMH11/19 | 0.016 | 0.25 | 0.75 | I | 0.008 | 2 | 0.75 | I | 2 | 4 | 8 | A | 1 | 16 | 6 | A | 1 | 4 | 2 | I |
| TMH12/19 | 0.004 | 0.064 | 1.064 | I | 0.016 | 8 | 6 | A | 2 | 4 | 8 | A | 1 | 16 | 6 | A | 0.5 | 8 | 2.5 | I |
| TMH13/19 | 0.004 | 0.125 | 0.75 | I | 0.004 | 8 | 0.75 | I | 1 | 4 | 10 | A | 0.25 | 16 | 1 | I | 0.5 | 32 | 2 | I |
| TMA13/20 | 0.008 | 0.125 | 1 | I | 0.004 | 4 | 0.75 | I | 0.5 | 0.5 | 9.81 | A | 0.064 | 8 | 2 | I | 0.125 | 16 | 2.5 | I |
| TMA14/20 | 0.08 | 0.032 | 0.506 | I | 0.008 | 4 | 0.75 | I | 0.5 | 0.25 | 9.81 | A | 0.064 | 4 | 1.5 | I | 0.125 | 16 | 3 | I |
| TMA15/20 | 0.016 | 0.032 | 0.506 | I | 0.008 | 8 | 0.375 | S | 0.125 | 0.125 | 2 | I | 0.064 | 8 | 0.762 | I | 0.125 | 16 | 1.5 | I |
| TMA16/20 | 0.008 | 0.25 | 0.625 | I | 0.008 | 8 | 0.375 | S | 0.25 | 0.5 | 2 | I | 0.125 | 8 | 0.75 | I | 0.5 | 32 | 2 | I |
| TMA6/19 | 0.008 | 0.125 | 0.75 | I | 0.004 | 2 | 0.75 | I | 0.5 | 2 | 8 | A | 0.5 | 8 | 6 | A | 1 | 8 | 4 | I |
| TMA7/19 | 0.004 | 0.125 | 0.625 | I | 0.004 | 16 | 0.75 | I | 1 | 8 | 10 | A | 0.25 | 16 | 0.75 | I | 0.5 | 32 | 1 | I |
| TMA16/19 | 0.032 | 0.5 | 1 | I | 0.016 | 16 | 0.75 | I | 0.5 | 2 | 9.81 | A | 0.032 | 16 | 0.75 | I | 1 | 32 | 2 | I |
| TMA1/19 | 0.004 | 0.25 | 0.5 | S | 0.004 | 4 | 0.75 | I | 0.125 | 1 | 2 | I | 0.032 | 4 | 0.756 | I | 0.5 | 4 | 1 | I |
| TMA28/17 | 0.032 | 2 | 5 | A | 0.008 | 4 | 0.75 | I | 2 | 2 | 12 | A | 0.032 | 4 | 0.756 | I | 0.5 | 8 | 2 | I |
| TMA18/19 | 0.008 | 0.5 | 0.75 | I | 0.008 | 4 | 0.75 | I | 0.25 | 4 | 9.81 | A | 0.032 | 8 | 1.5 | I | 2 | 32 | 3 | I |
| TMA19/19 | 0.016 | 1 | 0.75 | I | 0.008 | 8 | 0.25 | S | 0.5 | 2 | 2 | I | 0.25 | 16 | 0.75 | I | 1 | 32 | 1 | I |
| TMA21/17 | 0.004 | 0.25 | 0.75 | I | 0.004 | 2 | 0.75 | I | 0.5 | 4 | 12 | A | 0.032 | 4 | 0.756 | I | 1 | 16 | 3 | I |
| TMA31/18 | 0.008 | 0.125 | 0.75 | I | 0.008 | 4 | 0.75 | I | 0.5 | 2 | 10 | A | 0.064 | 8 | 1.012 | I | 1 | 16 | 3 | I |
| TMA23/17 | 0.004 | 0.064 | 0.628 | I | 0.004 | 1 | 0.75 | I | 0.5 | 1 | 9.81 | A | 0.125 | 16 | 5.95 | A | 0.25 | 8 | 2.5 | I |
| TMA24/17 | 0.004 | 0.25 | 0.75 | I | 0.004 | 2 | 0.75 | I | 0.5 | 4 | 11.81 | A | 0.016 | 4 | 0.756 | I | 2 | 16 | 4 | I |
| TMA12/19 | 0.016 | 0.25 | 5 | A | 0.008 | 4 | 0.75 | I | 2 | 0.5 | 12 | A | 1 | 32 | 6 | A | 0.125 | 16 | 2 | I |

| | | | | | | | | | | | | | | | | | | | |
|---|---|---|---|---|---|---|---|---|---|---|---|---|---|---|---|---|---|---|---|
| TMA13/19 | 0.064 | 1 | 5 | A | 0.125 | 64 | 5.9 | A | 2 | 2 | 10 | A | 0.25 | 15 | 0.75 | I | 0.25 | 16 | 2.5 | I |
| TMA25/17 | 0.008 | 0.064 | 0.756 | I | 0.008 | 4 | 0.75 | I | 0.5 | 1 | 8 | A | 0.125 | 8 | 1.5 | I | 0.5 | 16 | 3 | I |
| TMA9/19 | 0.004 | 0.25 | 0.75 | I | 0.016 | 16 | 6 | A | 0.5 | 2 | 9.81 | A | 0.125 | 16 | 5.95 | A | 0.5 | 8 | 2.5 | I |
| TMA10/19 | 0.004 | 0.064 | 0.628 | I | 0.004 | 2 | 0.75 | I | 2 | 1 | 10 | A | 0.125 | 8 | 1.5 | I | 0.5 | 8 | 2 | I |

Notes: I - Indifference, A – Antagonism, S – Synergism, TRB – terbinafine, ITC – itraconazole; EBs – ebselen; DPDS – diphenyl diselenide
